# Economic Growth and Childhood Malnutrition in Low- and Middle-Income Countries

**DOI:** 10.1001/jamanetworkopen.2023.42654

**Published:** 2023-11-09

**Authors:** Nicolas Büttner, Markus Heemann, Jan-Walter De Neve, Stéphane Verguet, Sebastian Vollmer, Kenneth Harttgen

**Affiliations:** 1 Department of Humanities, Social and Political Sciences, Eidgenössische Technische Hochschule Zürich, Zürich, Switzerland; 2 Department of Economics and Centre for Modern Indian Studies, University of Göttingen, Göttingen, Germany; 3 Heidelberg Institute of Global Health, Medical Faculty and University Hospital, Heidelberg University, Heidelberg, Germany; 4 Department of Global Health and Population, Harvard T. H. Chan School of Public Health, Boston, Massachusetts

## Abstract

**Importance:**

Economic growth may reduce childhood malnutrition through improvements of several contributing factors, but the empirical evidence is mixed. Identifying the most important factors that contribute to child malnutrition and their associations with economic growth can inform decision-making about targeted investments to improve children’s health.

**Objective:**

To assess the associations between economic growth and malnutrition, contributing factors and malnutrition, and economic growth and contributing factors of malnutrition in low- and middle-income countries (LMICs).

**Design, Setting, and Participants:**

This cross-sectional study used data from 239 Demographic and Health Surveys from January 1, 1990, to December 31, 2021. Observations included 1 138 568 children aged 0 to 35 months with valid anthropometric measures and information on contributing factors of malnutrition from 58 LMICs. Data were analyzed from May 20, 2022, to February 16, 2023.

**Exposure:**

National per-capita gross domestic product (GDP) was used as a proxy for economic growth.

**Main Outcomes and Measures:**

Six measures of childhood malnutrition were constructed: stunting (height-for-age *z* score <−2), underweight (weight-for-age *z* score <−2), wasting (weight-for-height *z* score <−2), overweight (weight-for-height *z* score >2), obesity (weight-for-height *z* score >3), and dietary diversity failure (consumption of less than 5 of 8 different food groups in the past 24 hours). Eighteen contributing factors of malnutrition were constructed, of which 10 were underlying determinants (eg, access to improved sanitation) and 8 were immediate determinants (eg, breastfeeding initiation).

**Results:**

A total of 1 138 568 children (mean [SD] age, 17.14 [10.26] months; 579 589 [50.9%] boys and 558 979 [49.1%] girls) were included in the analysis. Of these, 27.3% (95% CI, 27.2%-27.4%) had stunting; 25.7% (95% CI, 25.6%-25.8%), underweight; 11.2% (95% CI, 11.1%-11.2%), wasting; 3.8% (95% CI, 3.7%-3.8%), overweight; 1.1% (95% CI, 1.1%-1.1%), obesity; and 79.8% (95% CI, 79.7%-79.9%), dietary diversity failure. Per-capita GDP was weakly associated with childhood malnutrition. The odds ratios associated with a 5% increase in per-capita GDP were 0.99 (95% CI, 0.99-1.00) for stunting, 1.01 (95% CI, 1.00-1.01) for wasting, 1.00 (95% CI, 1.00-1.00) for underweight, 0.98 (95% CI, 0.98-0.98) for overweight, 0.98 (95% CI, 0.97-0.98) for obesity, and 1.03 (95% CI, 1.01-1.04) for dietary diversity failure. Although strong associations were found between many contributing factors and most outcomes for malnutrition, associations identified between per-capita GDP and these contributing factors themselves were ambiguous.

**Conclusions and Relevance:**

In this multicountry cross-sectional study, economic growth was weakly associated with childhood malnutrition and several contributing factors. To reduce child malnutrition, economic growth may need to be accompanied by more targeted investments to improve contributing factors that are strongly associated with child malnutrition, such as maternal health and education.

## Key Points

**Question:**

To what extent is economic growth associated with reduced prevalence of childhood malnutrition, what are the most important contributing factors of malnutrition, and how are these contributing factors associated with economic growth?

**Findings:**

This cross-sectional study of 1 138 568 children aged 0 to 35 months from 58 low- and middle-income countries found an ambiguous association between economic growth and the prevalence of childhood malnutrition. Moreover, while strong associations between several contributing factors and childhood malnutrition were identified, the associations between economic growth and these contributing factors themselves were often ambiguous.

**Meaning:**

These findings suggest that to reduce childhood malnutrition, economic growth should be accompanied by targeted investments that improve contributing factors of malnutrition not necessarily affected by economic growth, such as maternal human capital.

## Teaser

This cross-sectional study investigates the association between per-capita gross domestic product and children’s risk of being malnourished in low- and middle-income countries (LMICs) and evaluates contributing factors that provide the broadest scope for economic growth to reduce malnutrition.

## Introduction

Economic growth has been the focus of development policy for many national governments.^1^ Advocates of the trickle-down theories of macroeconomics argue that economic growth will automatically lead to a better quality of life and better health, including less childhood malnutrition.^2,3,4^ However, evidence suggests that economic growth has only a weak direct association with reductions in childhood stunting, wasting, and underweight.^5^ While the Sustainable Development Goals emphasize ending all forms of malnutrition by 2030, at the same time, there has been an alarming increase in childhood overweight and obesity in many emerging countries.^6^ This highlights the need for policy makers to better understand the factors that contribute to childhood malnutrition as well as the conditions that influence these factors.

**Figure 1.**
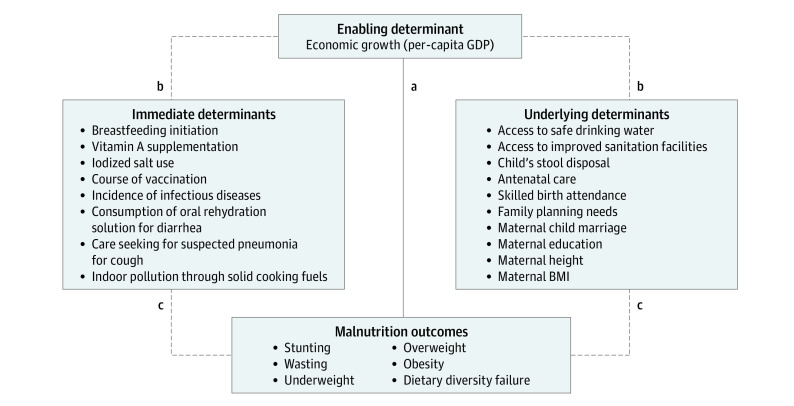
Conceptual Framework for Economic Growth and Child Malnutrition Most prior studies assess the indirect relationship between economic growth (per-capita gross domestic product [GDP]) and undernutrition, which can be interpreted as the total effect of economic growth on undernutrition (a). In the current study, we determined the direct association between economic growth and contributing factors (immediate and underlying determinants) (b) and the direct association between contributing factors (immediate and underlying determinants) and undernutrition (c). The effects (b and c) can be interpreted as the partial effects of economic growth on undernutrition. Additionally, we assess overnutrition and dietary diversity failure as further dimensions of malnutrition. Adapted from the 2020 United Nations Children’s Fund Conceptual Framework on Maternal and Child Nutrition.^9^ BMI indicates body mass index.

For this purpose, the United Nations Children’s Fund (UNICEF) developed a conceptual framework of malnutrition in 1990 as part of its Strategy for Improved Nutrition of Children and Women in Developing Countries.^7^ This framework has been continuously revised to “reflect advances in knowledge and priorities in child health and nutrition.”^8^ In 2020, UNICEF developed its new Conceptual Framework on the Determinants of Maternal and Child Nutrition, which builds on its initial 1990 framework but acknowledges the increasing triple burden of malnutrition, comprised of undernutrition, overnutrition, and micronutricient deficiencies, and further highlights the role of diets and care as immediate determinants of child nutrition.^9^ It describes the role of enabling, underlying, and immediate determinants in children’s and mothers’ nutritional outcomes. Enabling determinants include political, financial, social, cultural, and environmental conditions and influence children’s health through immediate and underlying determinants. Immediate determinants of malnutrition are more directly related to child malnutrition, like diets and care, while underlying determinants of malnutrition refer to family and community characteristics influencing a child’s well-being more indirectly (such as maternal education) (Figure 1).

Few studies have analyzed the association between aggregate economic growth and children’s risk of being malnourished. No evidence for such an association was found for India,^10^ while a negative association was found for Egypt.^11^ Two studies^5,12^ used large samples of pooled Demographic and Health Surveys (DHS) and found an inverse association between childhood stunting, underweight, and wasting and economic growth. One study^13^ found a large, inverse association between childhood stunting and economic growth based on pooled DHS data from 20 African countries. Empirical studies that investigate multiple household-level contributing factors of childhood malnutrition are also scarce. One recent cross-country study^14^ considered 9 direct and 17 indirect factors but analyzed only stunting, underweight, and wasting (omitting overweight, obesity, and dietary diversity failure). Other studies have focused on a specific country, like India^15^ and Kenya.^16^ A recent systematic review^17^ identified maternal educational level, household income, maternal nutrition, and access to sanitation as the most important contributing factors of childhood malnutrition.

In this study, we contribute to the understanding of the associations among economic growth, childhood malnutrition, and several potential contributing factors in low- and middle-income countries (LMICs). Combining individual-level data from 239 DHS from 58 LMICs, we first investigated the association between countries’ per-capita gross domestic product (GDP) and children’s risk of being malnourished. We built on work by Vollmer et al^5^ but used substantially more surveys and countries and considered overweight and obesity, as well as dietary diversity failure. We analyzed the associations of household-level contributing factors with malnutrition to test which contributing factors may provide the biggest scope for economic growth to reduce malnutrition. Last, we investigated the association between per-capita GDP and these contributing factors.

## Methods

### Ethics Clearance

This cross-sectional study was a complete case analysis. As DHS are publicly available anonymized data sets, this study was exempt from institutional review board approval and the need for informed consent based on national and institutional policies by ICF International, Inc. We complied with the Strengthening the Reporting of Observational Studies in Epidemiology (STROBE) reporting guideline.

### Data Sources

#### Malnutrition

Our data came primarily from the DHS, which have been conducted by ICF International Inc in more than 90 LMICs. The cross-sectional surveys use a multistage stratified sampling design and are nationally representative. They collect data regarding the health and welfare of women of reproductive age, their children, partners, and households. Additional details on the DHS are available elsewhere.^18^ We restricted our sample to surveys conducted between January 1, 1990, and December 31, 2021. Our sample was further restricted to countries with at least 2 surveys with all relevant data.

#### Economic Growth

Data on per-capita GDP were from the Penn World Tables, version 10.0, which provide national aggregate data for real per-capita GDP per year.^19^ Gross domestic product was adjusted for purchasing power parity to facilitate comparisons across countries. The aggregate GDP data were merged with the individual-level DHS data based on country and year.

### Study Population

We focused on children aged 0 to 35 months because in most DHS, anthropometric measurements are available only for children in this age group. Only children with complete data on malnutrition and contributing factors were included, yielding a maximum sample size of 1 138 568 children from 239 DHS surveys in 58 countries (eTable 1 in Supplement 1).

### Exposure

Our exposure was per-capita GDP at the country-year level. It was measured in logarithmic units to capture nonlinear associations with the outcomes. Gross domestic product was defined so that odds ratios (ORs) in logistic regression models correspond to a 5% increase in per-capita GDP.

### Outcomes

#### Childhood Malnutrition

Our primary outcome was individual-level childhood malnutrition, following the World Health Organization (WHO) 2006 Child Growth Standards.^20^ We considered binary indicators of stunting (1 if height-for-age *z* score <–2), wasting (1 if weight-for-height *z* score <–2), and underweight (1 if weight-for-age *z* score <–2). Overnutrition was measured by binary indicators of overweight (1 if weight-for-height *z* score >2) and obesity (1 if weight-for-height *z* score >3). For stunting, *z* scores were calculated as the child’s height minus the median height for that child’s age and sex in the WHO reference population, divided by the SD of this group in the reference population.^21^ The *z* scores for the other outcomes were calculated analogously. Biologically implausible values (defined by the WHO as, eg, a *z* score <−6 or >6 for height) were excluded.^20^ We also analyzed dietary diversity failure since anthropometric measures alone do not sufficiently capture malnutrition and should thus be complemented with dietary-based measures.^22^ This was measured as a binary indicator based on a score ranging from 0 to 8, with 1 point assigned for consuming grains, roots and tubers, legumes and nuts, dairy products, flesh foods, eggs, vitamin A–rich fruits, or vegetables in the past 24 hours before the interview. Scores lower than 5 indicated dietary diversity failure.

#### Potential Contributing Factors of Malnutrition

Our secondary outcomes were potential contributing factors of malnutrition (eTable 2 in Supplement 1). Here, we were conceptually guided by the 2020 UNICEF Conceptual Framework on Maternal and Child Nutrition and by existing empirical studies.^14^

As underlying determinants, we considered the following outcomes: no access to safe drinking water, no access to improved sanitation facilities, unsafe practices for child’s stool disposal, inadequate antenatal care, no skilled birth attendant, unsatisfied family planning needs, maternal child marriage, no maternal education, low maternal height, and low or high maternal BMI. As immediate determinants, we considered the following outcomes: delayed breastfeeding initiation, no vitamin A supplementation, no iodized salt use, incomplete course of vaccination, incidence of infectious diseases, no consumption of oral rehydration solution despite diarrhea, no care seeking for suspected pneumonia despite cough, and high indoor pollution through solid cooking fuels. All outcomes were coded as binary variables with the “better” category serving as the reference category.

### Control Variables

Household control variables included the household’s wealth quintile (with 5 being richest), size, and location (urban or rural). Child and mother control variables included the child’s sex, age, and birth order and the mother’s age at birth. Additionally, we added indicators for the countries to account for all observed and unobserved country-level factors (or above). We also controlled for the survey year to control for period effects and potential differences in measurement across time.

### Statistical Analysis

Data were analyzed from May 20, 2022, to February 16, 2023. Our main analysis consisted of 3 parts. First, we used logistic regression models to obtain ORs and 95% CIs for the association between per-capita GDP and our outcomes for child malnutrition, including undernutrition, overnutrition, and dietary diversity failure (Figure 1). Second, we regressed our outcomes for child malnutrition on potential contributing factors of child malnutrition, including immediate and underlying determinants (Figure 1). This analysis allowed us to assess which contributing factors provide the biggest scope for economic growth to reduce malnutrition. We estimated ORs using separate regression models for each malnutrition outcome but included all potential contributing factors as covariates. Here, we used only data from the latest available survey of each country and analyzed only the youngest child of the mother, as the associations between contributing factors and malnutrition likely changed over the study period. Third, we investigated the association between per-capita GDP and all potential contributing factors of malnutrition. To do so, we regressed each of these contributing factors of child malnutrition on per-capita GDP (Figure 1).

In all regressions with malnutrition as outcome, household, child, and mother control variables were included. In all regressions with contributing factors as outcomes, household control variables were included. We clustered SEs by primary sampling unit to account for intracluster correlation within primary sampling units, and we controlled for country fixed effects in all models to mitigate omitted variable bias. When using multiple DHS per country, that is, whenever per-capita GDP was the main regressor, we also included survey year fixed effects to control for global trends in GDP growth and our outcomes of interest.

To synthesize the importance of the various factors considered in our analyses, we used a Shorrocks decomposition.^23^ This method estimated the relative contribution of the different covariates to an outcome’s explained variance.

We classified associations based on the magnitude of ORs (in terms of distance from 1.00) obtained through the regression analyses. Large ORs indicated strong associations; small ORs, weak associations. We classified associations as ambiguous when ORs were generally close to 1.00 (weak) with some negative and some positive.

We conducted several sensitivity analyses based on a linear probability model. First, we reweighted the observations with the population size of the country using data from the United Nations population prospects.^24^ Second, we trimmed the sample to exclude extreme observations with regard to childhood malnutrition (1st, 2nd, 3rd, 98th, 99th, and 100th percentiles). Third, we used instrumental variable regressions with the investment share of GDP 5 years ago as an instrument for log per-capita GDP to address 2 potential statistical problems: measurement error in GDP that could bias the results downward, and endogeneity of GDP, which could bias the findings because of either reverse causality or omitted variable bias.

We used Stata, version 17.0 (StataCorp LLC) for all analyses. All statistical tests were 2 tailed, and *P* < .05 was chosen a priori to represent statistical significance.

## Results

### Sample Description

A total of 1 138 568 children (mean [SD] age, 17.14 [10.26] months; 579 589 [50.9%] boys and 558 979 [49.1%] girls) were included in the analysis. In the pooled sample, 27.3% (95% CI, 27.2%-27.4%) of children had stunting; 25.7% (95% CI, 25.6%-25.8%), underweight; 11.2% (95% CI, 11.1%-11.2%), wasting; 3.8% (95% CI, 3.7%-3.8%), overweight; 1.1% (95% CI, 1.1%-1.1%), obesity; and 79.8% (95% CI, 79.7%-79.9%), dietary diversity failure.

The Table provides summary statistics using aggregate country-level data from the latest DHS country (conducted between 1999 and 2021; median, 2018). There was substantial variation in per-capita GDP and malnutrition rates across countries and survey years. Per-capita GDP (purchasing power parity) ranged from US $785 in Burundi to US $23 118 in Turkey. Stunting ranged from 6.1% in Jordan to 42.3% in Burundi; wasting from 0.9% in Peru to 22.3% in Timor-Leste; underweight from 2.2% in Albania to 43.3% in Timor-Leste; overweight from 1.0% in Burundi to 12.6% in Morocco; obesity from 0.1% in Nepal to 4.2% in Morocco; and dietary diversity failure from 41.9% in Peru to 93.9% in Liberia.

**Table.** Summary of Study Variables^a^

Variables	No. of observations	No. of countries	Mean (SD) [range]
Indicators of malnutrition, %			
Stunting	320 502	57	21.1 (8.8) [6.1-42.3]
Wasting	320 736	57	6.9 (5.1) [0.9-22.3]
Underweight	320 223	57	18.2 (10.9) [2.2-43.3]
Overweight	320 736	57	4.2 (2.8) [1.0-12.6]
Obesity	320 736	57	1.2 (0.9) [0.1-4.2]
Dietary diversity failure	220 230	36	79.3 (11.8) [41.9-93.9]
Immediate determinants, %			
Delayed breastfeeding initiation	335 927	55	42.3 (16.8) [13.9-79.0]
No vitamin A supplementation	352 822	45	48.0 (16.7) [9.4-87.8]
No iodized salt use	203 911	16	21.1 (23.5) [4.4-93.5]
Incomplete course of vaccination	391 930	56	55.3 (14.6) [29.4-100.0]
Incidence of infectious diseases	365 718	57	51.7 (24.2) [12.4-100.0]
No consumption of oral rehydration solution despite diarrhea	391 259	55	10.7 (5.5) [1.2-26.9]
No care seeking for suspected pneumonia despite cough	377 784	56	8.2 (5.5) [1.3-29.7]
High indoor pollution through solid cooking fuels	335 950	47	70.8 (33.2) [0.0-99.9]
Underlying determinants, %			
No access to safe drinking water	369 172	57	23.2 (15.7) [1.1-61.1]
No access to improved sanitation facilities	365 187	57	41.0 (25.0) [0.0-88.6]
Unsafe practices for child’s stool disposal	269 374	41	50.9 (21.1) [9.6-84.6]
Inadequate antenatal care	338 202	57	34.1 (20.0) [2.3-74.8]
No skilled birth attendant	400 643	58	26.8 (21.5) [0.0-87.0]
Unsatisfied family planning needs	224 700	56	42.8 (20.7) [2.4-84.4]
Maternal child marriage	383 110	57	40.0 (17.3) [8.1-80.2]
No maternal education	380 505	58	26.4 (23.8) [0.0-85.3]
Low maternal height	325 219	56	3.4 (5.0) [0.1-28.9]
Low maternal BMI	301 183	56	8.9 (6.4) [0.4-28.9]
Per-capita GDP, US $			
Per-capita GDP in PPP	381 781	58	5195.06 (4723.97) [785.07-23 118.23]
5-y Lagged per-capita GDP in PPP	381 781	58	5117.01 (4657.50) [800.23-21 926.87]
Control variables			
Sex, %			
Female	381 781	58	49.3 (1.01) [46.2-51.8]
Male	381 781	58	50.7 (1.01) [48.2-53.8]
Child age, mo	381 781	58	17.00 (0.48) [15.95-18.19]
Birth order	379 931	58	1.09 (0.05) [1.00-1.17]
Maternal age at birth, y	381 781	58	26.89 (1.16) [23.55-29.92]
Urban, %	381 781	58	35.9 (14.7) [9.1-78.8]
Household size, No. of members	381 781	58	6.92 (1.88) [5.11-16.03]

Abbreviations: BMI, body mass index (calculated as weight in kilograms divided by height in meters squared); GDP, gross domestic product; PPP, purchasing power parity.

^a^ All statistics are based on the latest available Demographic and Health Surveys per country (conducted between 1999 and 2021). Sampling weights were used for aggregation at the country level.

### Association Between Economic Growth and Malnutrition

Per-capita GDP was negatively associated with stunting and positively with wasting, but for both only by a small magnitude (eTable 3 in Supplement 1). A 5% increase in per-capita GDP was associated with a 0.6% decrease in the odds of stunting (OR, 0.99 [95% CI, 0.99-1.00]; *P* < .001) and a 0.7% increase in the odds of wasting (OR, 1.01 [95% CI, 1.00-1.01]; *P* < .001). Per-capita GDP was not associated with underweight (OR, 1.00 [95% CI, 1.00-1.00]). In contrast, associations were found for overnutrition. A 5.0% increase in per-capita GDP was associated with a 1.9% decrease in the odds of having overweight (OR, 0.98 [95% CI, 0.98-0.98]; *P* < .001) and a 2.4% decrease for having obesity (OR, 0.98 [95% CI, 0.97-0.98]; *P* < .001). Last, a 5.0% increase in per-capita GDP was associated with a 2.5% increase in the odds of dietary diversity failure (OR, 1.03 [95% CI, 1.01-1.04]; *P* < .001). Except for wasting, the results were robust to using linear probability models, to weighting observations by countries’ population size, and to excluding outliers. Only overweight and obesity were robust to the instrumental variable specification (eTable 4 in Supplement 1).

### Association Between Contributing Factors and Malnutrition

We here show the results for 1 indicator per category, that is, stunting for undernutrition, overweight for overnutrition, and dietary diversity failure. The full results are shown in eFigure 1 in Supplement 1. To maximize sample size, the main specification did not include the covariates of unsafe practices for child’s stool disposal, no vitamin A supplementation, and no iodized salt use. The results from regressions including these covariates are shown in eFigure 2 in Supplement 1.

#### Potential Contributing Factors and Undernutrition

**Figure 2.**
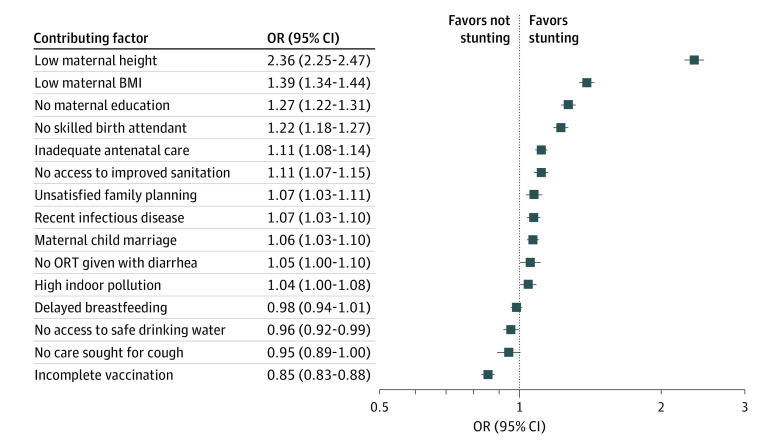
Associations Between Contributing Factors and Stunting Includes 135 310 participants in 56 countries. Squares represent point estimates of odds ratios (ORs) associated with stunting. Contributing factors include immediate and underlying determinants of malnutrition that were available in the Demographic and Health Surveys. All specifications include country and survey year fixed effects as well as household, child, and mother control variables. Error bars indicate 95% CIs. BMI indicates body mass index (calculated as weight in kilograms divided by height in meters squared); ORT, oral rehydration therapy.

Figure 2 shows the ORs for stunting associated with 15 contributing factors (eFigure 1 in Supplement 1 for underweight and wasting). Nine of 15 analyzed covariates had positive and significant ORs. With an OR of 2.36 (95% CI, 2.25-2.47), low maternal height shows the strongest association, followed by low maternal BMI (1.39 [95% CI, 1.34-1.44), no maternal education (1.27 [95% CI, 1.22-1.31]), no skilled birth attendant (1.22 [95% CI, 1.18-1.27]), inadequate antenatal care (1.11 [95% CI, 1.08-1.14]), and no access to improved sanitation facilities (OR, 1.11 [95% CI, 1.07-1.15]). Last, unsatisfied family planning needs (1.07 [95% CI, 1.03-1.11]), the incidence of infectious diseases (1.07 [95% CI, 1.03-1.10]), and maternal child marriage (1.06 [95% CI, 1.03-1.10]) increased the odds of stunting. We found no associations with lack of consumption of oral rehydration solution despite diarrhea, high indoor pollution through solid cooking fuels, delayed breastfeeding initiation, and no care seeking for suspected pneumonia despite cough. Against our expectation, we found negative associations with no access to safe drinking water (0.96 [95% CI, 0.92-0.99]) and incomplete course of vaccination (0.85 [95% CI, 0.83-0.88]). The latter was driven by vaccinations against measles, which was more common among children with stunting. In India, which contributed 29% of children in the sample, the measles vaccination rate was approximately 10 percentage points higher among children with stunting. All results were robust to using linear probability models and to excluding outliers. All covariates except no skilled birth attendant and maternal child marriage were also robust to weighting by population size (eTable 5 in Supplement 1).

The results for underweight had comparable ORs for most covariates (eFigure 1 in Supplement 1). Notably, the OR for low maternal BMI was markedly larger (1.90 [95% CI, 1.83-1.97]), and the ORs for no consumption of oral rehydration solution despite diarrhea (1.07 [95% CI, 1.01-1.13]) and high indoor pollution through solid cooking fuels (1.05 [95% CI, 1.01-1.10]) became significant. The results were robust to using linear probability models, to excluding outliers (except no consumption of oral rehydration solution despite diarrhea), and to weighting by population size (except unsatisfied family planning needs, no consumption of oral rehydration solution despite diarrhea, and high indoor pollution through solid cooking fuels). No access to safe drinking water turned positive in all 3 sensitivity tests (eTable 5 in Supplement 1). The results for wasting were also similar, yet the ORs were much smaller and less precisely estimated (eFigure 1 in Supplement 1).

#### Potential Contributing Factors and Overnutrition

**Figure 3.**
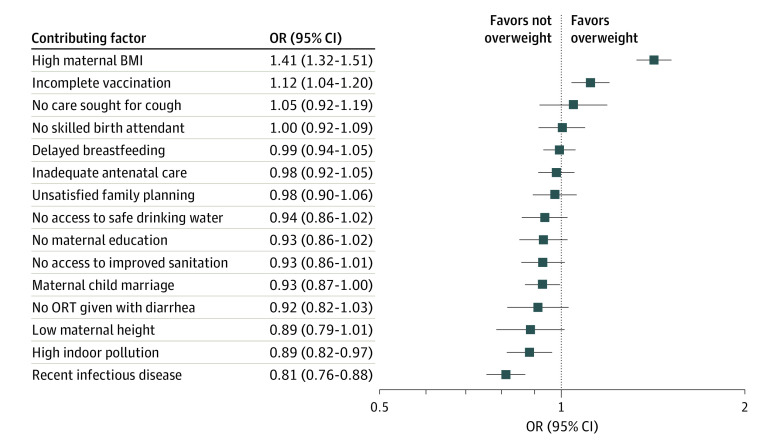
Associations Between Contributing Factors and Overweight Includes 135 669 participants in 56 countries. Squares represent point estimates of odds ratios (ORs) associated with overweight. Contributing factors include immediate and underlying determinants of malnutrition that were available in the Demographic and Health Surveys. All specifications include country and survey year fixed effects as well as household, child, and mother control variables. Error bars indicate 95% CIs. BMI indicates body mass index (calculated as weight in kilograms divided by height in meters squared); ORT, oral rehydration therapy.

For overweight, positive and significant ORs were only found with high maternal BMI (1.41 [95% CI, 1.32-1.51]) and incomplete course of vaccination (1.12 [95% CI, 1.04-1.20]), while for most remaining covariates, ORs were either insignificant or negative and of small to moderate magnitude (Figure 3). A similar pattern was found for obesity. For both outcomes, the positive associations with high maternal BMI and incomplete course of vaccination remain robust to all sensitivity tests (eTable 5 in Supplement 1).

#### Potential Contributing Factors and Dietary Diversity Failure

**Figure 4.**
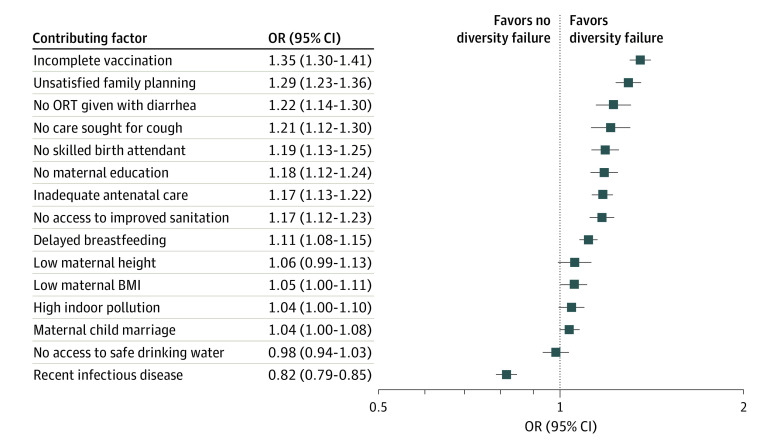
Associations Between Contributing Factors and Dietary Diversity Failure Includes 98 842 participants in 51 countries. Squares represent point estimates of odds ratios (ORs) associated with dietary diversity failure. Contributing factors include immediate and underlying determinants of malnutrition that were available in the Demographic and Health Surveys. All specifications include country and survey year fixed effects as well as household, child, and mother control variables. Error bars indicate 95% CIs. BMI indicates body mass index (calculated as weight in kilograms divided by height in meters squared); ORT, oral rehydration therapy.

Dietary diversity failure was positively associated with 10 of 15 contributing factors, most strongly with incomplete course of vaccination (OR, 1.35 [95% CI, 1.30-1.41]), followed by unsatisfied family planning needs (OR, 1.29 [95% CI, 1.23-1.36]), no consumption of oral rehydration solution despite diarrhea (OR, 1.22 [95% CI, 1.14-1.30]), no care seeking for suspected pneumonia despite cough (OR 1.21 [95% CI, 1.12-1.30]), no skilled birth attendant (OR, 1.19 [95% CI, 1.13-1.25]), no maternal education (OR, 1.18 [95% CI, 1.12-1.24]), inadequate antenatal care (OR. 1.17 [95% CI, 1.13-1.22]), no access to improved sanitation facilities (OR, 1.17 [95% CI, 1.12-1.23]), delayed breastfeeding initiation (OR, 1.11 [95% CI, 1.08-1.15]), and low maternal BMI (OR, 1.05 [95% CI, 1.00-1.11]) (Figure 4). These results were robust to all sensitivity tests (eTable 5 in Supplement 1).

### Association Between Economic Growth and Contributing Factors

eFigure 3 in Supplement 1 shows ORs for immediate and underlying determinants corresponding to a 5% increase in per-capita GDP. The theoretical framework suggested a negative association between economic growth and contributing factors, but this was not unanimously confirmed. Among immediate determinants, sizable negative associations with per-capita GDP were only found for no iodized salt use (OR, 0.95 [95% CI, 0.95-0.95]) and high indoor pollution through solid cooking fuels (OR, 0.96 [95% CI, 0.96-0.96]), both robust to all sensitivity tests except for instrumental variable regressions (eTable 6 in Supplement 1). Small negative associations were found for no vitamin A supplementation (OR, 0.99 [95% CI, 0.99-1.00]) and no consumption of oral rehydration solution despite diarrhea (OR, 0.99 [95% CI, 0.99-1.00]). The remaining immediate determinants were positively associated or not associated with per-capita GDP.

We found negative associations between most underlying determinants and per-capita GDP. Apart from unsafe practices for child’s stool disposal (OR, 0.97 [95% CI, 0.97-0.97]), all ORs were very small. No access to safe drinking water and unsatisfied family planning needs were positively associated with per-capita GDP. Unsafe practices for child’s stool disposal and access to improved sanitation were robust to all sensitivity tests, and inadequate antenatal care, low maternal height, and low maternal BMI were robust to all except instrumental variable regressions (eTable 6 in Supplement 1).

### Shorrocks Decomposition

The Shorrocks decompositions showed for all malnutrition outcomes a very small contribution of per-capita GDP to the pseudo-*R*^2^ value of our logit regressions. Per-capita GDP explains between 1% and 7% of the pseudo-*R*^2^ value (eTable 7 in Supplement 1). It also explains a very limited share of the variance in contributing factors (eTable 9 in Supplement 1). However, with 19% to 38%, all contributing factors together explain a nonnegligible share of the variance in malnutrition outcomes. Further, the Shorrocks decompositions show a very large contribution of the country fixed effects to the explained variation in the outcome (eTables 6-8 in Supplement 1). Country fixed effects contribute 29% to 67% (eTable 7 in Supplement 1), 32% to 76% (eTable 8 in Supplement 1), and 31% to 82% (eTable 9 in Supplement 1) of explained variation in the outcomes. This highlights the importance of country-specific (but unobserved) factors for malnutrition prevalence and contributing factors. Differences in the disease environment, the health system, and socioeconomic conditions vary across countries and need to be considered in the design and implementation of interventions tackling child malnutrition.

## Discussion

Our results in this cross-sectional study reveal 3 salient findings. First, we found a weak association between economic growth and child undernutrition, which contrasts with the expectations from the framework, but is in line with previous empirical findings.^5,12^ For overnutrition, we found a negative association, and for dietary diversity failure a positive association. This positive association could partly be driven by the increasing availability of ultraprocessed foods in higher-income countries,^25^ which could contribute to the increasing double burden of undernutrition and overnutrition already observed in many emerging countries.^26^ Second, our results confirm a strong link between several contributing factors of malnutrition and our outcomes for undernutrition.^14^ Third, we found that economic growth was ambiguously associated with most contributing factors of malnutrition. Economic growth was not associated with maternal education, for example, although maternal education itself was strongly linked with malnutrition. Investments in education, especially targeted to girls, may contribute to significant reductions in child malnutrition in certain contexts.^27^

There are several potential reasons why economic growth does not automatically reduce child undernutrition.^5^ First, income growth could be unequally distributed, and poor households might not benefit substantially from increases in aggregate per-capita GDP. Second, they might not necessarily invest potential additional income into improving their children’s nutrition. Third, income growth does not necessarily lead to investments that help improve children’s nutritional status. We provided empirical evidence for the relevance of these channels by showing that indeed, increases in per-capita GDP are only weakly associated with households’ health-related behavior or access to public services.

Given the large number of countries and the long period covered in our analyses, it is important to note that our results show mean associations. The true associations will vary across countries, time periods, and even among different population subgroups, which is crucial to consider in the design of public health interventions.

Our results do not question the validity of the UNICEF Conceptual Framework on the Determinants of Maternal and Child Nutrition. Instead, they underline 2 important aspects in the association between economic growth and child malnutrition. First, many contributing factors, such as maternal education and health, are important in combating child malnutrition. Second, aggregate economic growth may not automatically improve these household-level determinants. This is in line with the framework that includes as enabling factors, next to financial resources, political, social, cultural, and environmental conditions, which may affect if and how financial resources are used to alleviate childhood malnutrition in LMICs.

### Limitations

This study has some limitations. The first concerns the measurement of undernutrition, in particular underweight and wasting. Improvements in these outcomes might not necessarily indicate improved nutritional status but could also mirror a change in diet toward more sugar and animal fats.^28^ However, this issue is less relevant for stunting, and our results are similar for all 3 measures of undernutrition. In addition, genetic differences across populations from different countries might bias the results,^28^ yet we mitigate this concern by including country fixed effects. Further issues are related to the sampling and quality of DHS and Penn World Tables. The DHS data sets cover disproportionately fewer very poor countries, limiting external validity for countries not included in our sample. Finally, as explained in detail elsewhere,^5^ reverse causality could be a source of bias. First, children’s nutritional status could negatively affect per-capita GDP (eg, by reducing parents’ labor hours when caring for malnourished children). Second, children’s nutritional status could be a proxy for overall population health, which affects the economic development of a country.^3,29,30^ Concerns of potential reverse causality and unobserved heterogeneity were mitigated through instrumental variable regressions (eTables 3 and 5 in Supplement 1).^5^

## Conclusions

In this cross-sectional study, economic growth was weakly associated with childhood malnutrition and several contributing factors. Economic growth should not be considered a sufficient condition to improve childhood malnutrition but should be accompanied by targeted investments to improve important contributing factors, such as the human capital of mothers, providing them with increased opportunities to reduce malnutrition among their children.

## Notes

Supplement 1. **eTable 1.** Number of Surveys and Most Recent Survey Year per Country **eTable 2.** Definition of Contributing Factors of Malnutrition **eTable 3.** Adjusted Odds Ratios for Childhood Malnutrition Associated With the Log of Per-Head GDP **eTable 4.** Association Between Economic Growth and Malnutrition—Sanity Tests **eTable 5.** Association Between Determinants and Indicators of Malnutrition—Sanity Tests **eTable 6.** Association Between Economic Growth and Determinants of Malnutrition—Sanity Tests **eTable 7.** Association Between Economic Growth and Malnutrition—Shorrocks Decomposition **eTable 8.** Association Between Determinants and Indicators of Malnutrition—Shorrocks Decomposition **eTable 9.** Association Between Economic Growth and Determinants of Malnutrition—Shorrocks Decomposition **eFigure 1.** Association Between Determinants and Indicators of Malnutrition—Standard Model (15 Determinants) **eFigure 2.** Association Between Determinants and Indicators of Malnutrition—Extended Model (18 Determinants) **eFigure 3.** Associations Between Per-Head GDP and Contributing Factors of Malnutrition

Supplement 2.
Data Sharing Statement
