# Supplementary material for: Economic Growth and Childhood Malnutrition in Low- and Middle-Income Countries
Source: JAMA Netw Open. 2023 Nov 9;6(11):e2342654. doi: 10.1001/jamanetworkopen.2023.42654 (PMC10636637; doi:10.1001/jamanetworkopen.2023.42654)
Supplement: Supplement 1. — eTable 1. Number of Surveys and Most Recent Survey Year per Country eTable 2. Definition of Contributing Factors of Malnutrition eTable 3. Adjusted Odds Ratios for Childhood Malnutrition Associated With the Log of Per-Head GDP eTable 4. Association Between Economic Growth and Malnutrition—Sanity Tests eTable 5. Association Between Determinants and Indicators of Malnutrition—Sanity Tests eTable 6. Association Between Economic Growth and Determinants of Malnutrition—Sanity Tests eTable 7. Association Between Economic Growth and Malnutrition—Shorrocks Decomposition eTable 8. Association Between Determinants and Indicators of Malnutrition—Shorrocks Decomposition eTable 9. Association Between Economic Growth and Determinants of Malnutrition—Shorrocks Decomposition eFigure 1. Association Between Determinants and Indicators of Malnutrition—Standard Model (15 Determinants) eFigure 2. Association Between Determinants and Indicators of Malnutrition—Extended Model (18 Determinants) eFigure 3. Associations Between Per-Head GDP and Contributing Factors of Malnutrition [file jamanetwopen-e2342654-s001.pdf]

Supplementary Online Content

Büttner N, Heemann M, De Neve JW, Verguet S, Vollmer S, Harttgen K. Economic growth and childhood malnutrition in low- and middle-income countries. *JAMA Netw Open*. 2023;6(11):e2342654. doi:10.1001/jamanetworkopen.2023.42654

eTable 1. Number of Surveys and Most Recent Survey Year per Country

eTable 2. Definition of Contributing Factors of Malnutrition

eTable 3. Adjusted Odds Ratios for Childhood Malnutrition Associated With the Log of Per-Head GDP

eTable 4. Association Between Economic Growth and Malnutrition—Sanity Tests

eTable 5. Association Between Determinants and Indicators of Malnutrition—Sanity Tests

eTable 6. Association Between Economic Growth and Determinants of Malnutrition—Sanity Tests

eTable 7. Association Between Economic Growth and Malnutrition—Shorrocks Decomposition

eTable 8. Association Between Determinants and Indicators of Malnutrition—Shorrocks Decomposition

eTable 9. Association Between Economic Growth and Determinants of Malnutrition—Shorrocks Decomposition

eFigure 1. Association Between Determinants and Indicators of Malnutrition—Standard Model (15 Determinants)

Figure 2. Association Between Determinants and Indicators of Malnutrition—Extended Model (18 Determinants)

eFigure 3. Associations Between Per-Head GDP and Contributing Factors of Malnutrition

This supplementary material has been provided by the authors to give readers additional information about their work.

© 2023 Büttner N et al. *JAMA Network Open*.

eTable 1. Number of Surveys and Most Recent Survey Year per Country

Country	Number of surveys	Most recent survey year
Albania	2	2018
Angola	3	2015
Armenia	4	2016
Bangladesh	5	2014
Benin	5	2018
Bolivia	2	2008
Burkina Faso	4	2010
Burundi	3	2017
Cambodia	4	2014
Cameroon	5	2018
Chad	3	2015
Colombia	4	2015
Comoros	2	2012
Congo	2	2012
Congo Democratic Republic	2	2014
Cote d'Ivoire	3	2012
Dominican Republic	6	2013
Egypt	5	2014
Ethiopia	5	2019
Gabon	2	2012
Gambia	2	2020
Ghana	6	2016
Guatemala	3	2015
Guinea	5	2021
Haiti	5	2017
Honduras	2	2012
India	3	2020
Jordan	5	2018
Kazakhstan	2	1999
Kenya	5	2020
Kyrgyz Republic	2	2012
Lesotho	3	2014
Liberia	6	2020
Madagascar	7	2016
Malawi	7	2017
Maldives	2	2017
Mali	5	2018
Morocco	2	2004
Mozambique	5	2018
Namibia	4	2013
Nepal	3	2016
Nicaragua	2	2001
Niger	4	2012
Nigeria	7	2018
Pakistan	2	2018
Peru	8	2012
Rwanda	7	2020
Senegal	11	2019
Sierra Leone	4	2019
Tajikistan	2	2017
Tanzania	9	2017
Timor-Leste	2	2016
Togo	3	2017
Turkey	3	2013
Uganda	7	2016
Yemen	2	2013
Zambia	6	2018
Zimbabwe	5	2015

© 2023 Büttner N et al. *JAMA Network Open*.

eTable 2. Definition of Contributing Factors of Malnutrition

Contributing factor	Description
Enabling Environments	
No access to safe drinking water	=1 if household does not have access to water piped into dwelling, yard or plot, public tab of standpipe, tube well or borehole, or protected well
No access to improved sanitation facilities	=1 if household does not have access to flush toilet, septic tank, or pit latrine
Unsafe practices for child's stool disposal	=1 if child does not use toilet/latrine, if fecal matter is not disposed by toilet/latrine, disposable diaper or burying
Inadequate antenatal care	=1 if mother had less than 4 antenatal care visits from a skilled provider for most recent birth
No skilled birth attendant	=1 if child was delivered without skilled birth attendants (e.g., physicians, nurses, midwives)
Unsatisfied family planning needs	=1 if mother wishes to stop/delay childbearing, but is not using any modern contraception method
Maternal child marriage	=1 if mother was married before age 18
No maternal education	=1 if mother has no formal education
Low maternal height	=1 if mother's height is less than 145cm/155cm
Low/high maternal BMI	=1 if mother's BMI is less than 18·5 / more than 25·0
Proximal Components	
Delayed breastfeeding initiation	=1 if mother started breastfeeding of child later than one hour after birth
No vitamin A supplementation	=1 if child did not receive vitamin A supplement in past 6 months
No iodized salt use	=1 if household does not use iodized salt
Incomplete course of vaccination	=1 if child is not fully vaccinated against all following diseases: tuberculosis, diphtheria, pertusis, tetanus, polia, measles
Incidence of infectious diseases	=1 if child had diarrhea, cough, or fever in the past 2 weeks
No consumption of oral rehydration solution despite diarrhea	=1 if child had diarrhea recently but did not receive oral rehydration therapy
No care seeking for suspected pneumonia despite cough	=1 if child had cough in past two weeks and no treatment was sought
High indoor pollution through solid cooking fuels	=1 if household uses solid cooking fuels (e.g., coal/lignite, charcoal, wood, straw/shrub/grass, agricultural crops, animal dung)

BMI=body mass index (calculated as weight in kilograms divided by height in meters squared).

© 2023 Büttner N et al. *JAMA Network Open*.

eTable 3. Adjusted Odds Ratios for Childhood Malnutrition Associated With the Log of Per-Head GDP

	Malnutrition					
	Undernutrition			Overnutrition		Dietary diversity failure
**Log of per-head GDP**	Stunting	Wasting	Underweight	Overweight	Obesity	
**OR**	0·994	1·007	1·000	0·981	0·976	1·025
**95% CI**	[0·992–0·996]	[1·003–1·010]	[0·998–1·002]	[0·979–0·984]	[0·973–0·980]	[1·014–1·039]
**p-value**	<0·001	<0·001	0·734	<0·001	<0·001	<0·001
**N**	899,531	900,246	896,593	900,246	900,246	536,649
**Countries**	57	57	57	57	57	36
**Years**	1990–2020	1990–2020	1990–2020	1990–2020	1990–2020	2005–2020

Data for the per-head gross domestic product (GDP) were merged with Demographic and Health Survey data by survey-year. Standard errors are clustered at the primary sampling unit level. Odds ratios (ORs) for the log of per-head GDP represent the difference in odds associated with a 5% increase in per-head GDP. All specifications include country and survey-year fixed effects as well as household and child/mother control variables. All ORs are rounded to three decimal places; thus, an OR of 1·000 in the confidence interval (CI) does not necessarily imply that the value 1 is included in the CI.

© 2023 Büttner N et al. *JAMA Network Open*.

eTable 4. Association Between Economic Growth and Malnutrition—Sanity Tests

	Stunting	Wasting	Under-weight	Over-weight	Obesity	Dietary Diversity Failure
**Unweighted**						
Log of per-head GDP	-0·0011	0·0005	0·0001	-0·0011	-0·0006	0·0033
(SE)	(0·0002)	(0·0001)	(0·0002)	(0·0001)	(0·0001)	(0·0005)
p value	<0·001	<0·001	0·766	<0·001	<0·001	<0·001
Number of observations	899,531	900,246	896,593	900,246	900,246	536,649
**Weighted**						
Log of per-head GDP	-0·0027	0·0003	0·0002	-0·0025	-0·0012	0·0040
(SE)	(0·0003)	(0·0002)	(0·0003)	(0·0002)	(0·0002)	(0·0005)
p value	<0·001	0·131	0·400	<0·001	<0·001	<0·001
Number of observations	899,531	900,246	896,593	900,246	900,246	536,649
**Trimmed**						
Log of per-head GDP	-0·0010	-0·0002	0·0001	-0·0002	-0·0001	0·0032
(SE)	(0·0002)	(0·0001)	(0·0002)	(0·0001)	(0·0001)	(0·0006)
p value	<0·001	0·215	0·654	0·060	0·034	<0·001
Number of observations	871,913	683,221	845,741	857,824	865,145	520,930
**Instrumental Variable**						
Log of per-head GDP	-0·0133	0·0040	0·0394	-0·0219	-0·0096	-0·0091
(SE)	(0·0083)	(0·0052)	(0·0107)	(0·0042)	(0·0021)	(0·0024)
p value	0·111	0·434	<0·001	<0·001	<0·001	<0·001
Number of observations	899,009	899,685	896,071	899,685	899,685	536,649

Data for the per-head gross domestic product (GDP) were merged with Demographic and Health Survey data by survey year. All regressions are ordinary least squares, and the instrumental variable regressions are two-stage least squares. All specifications include country and survey-year fixed effects as well as household and child/mother control variables. SEs are clustered at the PSU level. Coefficients for the log of per-head GDP represent a 5% increase in per-head GDP. In the instrumental variable regressions, we used the variable share of gross capital formation at present purchasing power parity (investment share of GDP) from the Penn World Tables 10.0, with a 5-year lag as an instrument for the log of the per-head GDP.

© 2023 Büttner N et al. *JAMA Network Open*.

eTable 5. Association Between Determinants and Indicators of Malnutrition—Sanity Tests

	Stunting			Wasting			Underweight		
	**Un-weigh-ted**	**Weigh-ted**	**Trim-med**	**Un-weigh-ted**	**Weigh-ted**	**Trim-med**	**Un-weigh-ted**	**Weigh-ted**	**Trim-med**
**Delayed breastfeeding**	-0·002	0·001	-0·003	0·003	-0·001	0·002	0·005	0·000	0·004
**(SE)**	(0·002)	(0·004)	(0·002)	(0·002)	(0·003)	(0·002)	(0·002)	(0·004)	(0·002)
**p value**	0·313	0·847	0·249	0·107	0·598	0·184	0·046	0·986	0·069
**Incomplete vaccination**	-0·022	-0·016	-0·021	-0·016	-0·021	-0·016	-0·026	-0·028	-0·026
**(SE)**	(0·003)	(0·005)	(0·003)	(0·002)	(0·003)	(0·002)	(0·003)	(0·005)	(0·003)
**p value**	<0·001	0·001	<0·001	<0·001	<0·001	<0·001	<0·001	<0·001	<0·001
**Recent infectious**	0·010	0·017	0·009	0·015	0·017	0·015	0·032	0·038	0·032
**disease (SE)**	(0·003)	(0·005)	(0·003)	(0·002)	(0·003)	(0·002)	(0·003)	(0·005)	(0·003)
**p value**	0·001	<0·001	0·002	<0·001	<0·001	<0·001	<0·001	<0·001	<0·001
**No ORT given with**	0·007	0·013	0·006	-0·000	-0·006	-0·001	0·008	0·015	0·008
**diarrhea (SE)**	(0·004)	(0·008)	(0·004)	(0·003)	(0·006)	(0·003)	(0·004)	(0·009)	(0·004)
**p value**	0·098	0·125	0·149	0·902	0·263	0·863	0·050	0·072	0·065
**No care sought for cough**	-0·011	-0·012	-0·010	-0·009	-0·000	-0·009	-0·014	-0·003	-0·013
**(SE)**	(0·005)	(0·009)	(0·005)	(0·003)	(0·007)	(0·003)	(0·004)	(0·009)	(0·004)
**p value**	0·015	0·199	0·030	0·004	0·956	0·003	0·002	0·766	0·003
**High indoor pollution**	-0·008	0·005	-0·009	-0·009	-0·004	-0·008	-0·008	-0·003	-0·008
**(SE)**	(0·003)	(0·006)	(0·003)	(0·002)	(0·004)	(0·002)	(0·003)	(0·006)	(0·003)
**p value**	0·014	0·426	0·011	0·000	0·328	<0·001	0·014	0·638	0·012
**No access safe drinking**	0·019	0·014	0·019	0·008	0·002	0·008	0·026	0·018	0·026
**water (SE)**	(0·003)	(0·005)	(0·003)	(0·002)	(0·004)	(0·002)	(0·003)	(0·005)	(0·003)
**p value**	<0·001	0·005	<0·001	0·001	0·664	0·001	<0·001	0·001	<0·001
**No access improved**	0·018	0·024	0·018	0·001	0·003	0·001	0·016	0·016	0·016
**sanitation (SE)**	(0·003)	(0·004)	(0·003)	(0·002)	(0·003)	(0·002)	(0·003)	(0·004)	(0·003)
**p value**	<0·001	<0·001	<0·001	0·523	0·359	0·461	<0·001	<0·001	<0·001
**Inadequate antenatal**	0·038	0·034	0·038	0·004	0·009	0·004	0·023	0·031	0·023
**care (SE)**	(0·004)	(0·006)	(0·004)	(0·002)	(0·004)	(0·003)	(0·003)	(0·006)	(0·003)
**p value**	<0·001	<0·001	<0·001	0·109	0·041	0·121	0·000	0·000	0·000
**No skilled birth attendant**	0·011	0·009	0·012	0·004	0·015	0·004	0·013	0·018	0·013
**(SE)**	(0·003)	(0·006)	(0·003)	(0·002)	(0·004)	(0·002)	(0·003)	(0·006)	(0·003)
**p value**	0·001	0·147	<0·001	0·072	<0·001	0·066	<0·001	0·005	<0·001
**Unsatisfied family**	0·011	0·010	0·011	0·002	0·004	0·002	0·011	0·007	0·010
**planning (SE)**	(0·003)	(0·004)	(0·003)	(0·002)	(0·003)	(0·002)	(0·002)	(0·004)	(0·003)
**p value**	<0·001	0·020	<0·001	0·197	0·150	0·305	<0·001	0·089	<0·001
**Maternal child marriage**	0·010	0·010	0·010	0·007	0·006	0·006	0·017	0·017	0·016
**(SE)**	(0·003)	(0·006)	(0·003)	(0·003)	(0·004)	(0·003)	(0·003)	(0·006)	(0·003)
**p value**	0·002	0·085	0·003	0·013	0·155	0·020	<0·001	0·005	<0·001
**No maternal education**	0·049	0·060	0·049	0·015	0·018	0·015	0·053	0·059	0·053
**(SE)**	(0·004)	(0·005)	(0·004)	(0·003)	(0·004)	(0·003)	(0·003)	(0·005)	(0·003)
**p value**	<0·001	<0·001	<0·001	<0·001	<0·001	<0·001	<0·001	<0·001	<0·001
**Low maternal height**	0·178	0·168	0·179	0·005	0·006	0·005	0·129	0·136	0·131
**(SE)**	(0·005)	(0·008)	(0·005)	(0·004)	(0·005)	(0·004)	(0·005)	(0·007)	(0·005)
**p value**	<0·001	<0·001	<0·001	0·202	0·238	0·215	<0·001	<0·001	<0·001
**Low maternal BMI**	0·066	0·064	0·068	0·057	0·054	0·057	0·135	0·134	0·136
**(SE)**	(0·004)	(0·006)	(0·004)	(0·003)	(0·004)	(0·003)	(0·004)	(0·006)	(0·004)
**p value**	<0·001	<0·001	<0·001	<0·001	<0·001	<0·001	<0·001	<0·001	<0·001
**Number of observations**	135,310	135,310	131,580	135,669	135,669	131,506	135,567	135,567	131,837

Part 1

BMI=body mass index (calculated as weight in kilograms divided by height in meters squared). ORT=oral rehydration therapy. All regressions are ordinary least squares. All specifications are ordinary least squares and include country-fixed effects as well as household and child/mother control variables. SEs are clustered at the PSU level.

© 2023 Büttner N et al. *JAMA Network Open*.

	Overweight			Obesity			Dietary Diversity Failure		
	Un-weigh-ted	Weigh-ted	Trim-med	Un-weigh-ted	Weigh-ted	Trim-med	Un-weigh-ted	Weigh-ted	Trim-med
**Delayed breastfeeding**	0·000	-0·000	0·000	-0·001	-0·001	-0·001	0·015	0·009	0·015
**(SE)**	(0·001)	(0·001)	(0·001)	(0·001)	(0·001)	(0·001)	(0·003)	(0·004)	(0·003)
**p value**	0·921	0·781	0·909	0·057	0·121	0·068	0·000	0·015	<0·001
**Incomplete vaccination**	0·009	0·008	0·009	0·003	0·004	0·003	0·051	0·033	0·050
**(SE)**	(0·001)	(0·001)	(0·001)	(0·001)	(0·001)	(0·001)	(0·003)	(0·005)	(0·004)
**p value**	<0·001	<0·001	<0·001	<0·001	<0·001	<0·001	<0·001	<0·001	<0·001
**Recent infectious**	-0·008	-0·009	-0·009	-0·003	-0·003	-0·003	-0·028	-0·019	-0·028
**disease(SE)**	(0·001)	(0·002)	(0·001)	(0·001)	(0·001)	(0·001)	(0·003)	(0·005)	(0·003)
**p value**	<0·001	<0·001	<0·001	<0·001	0·004	<0·001	<0·001	<0·001	<0·001
**No ORT given with**	-0·003	-0·004	-0·004	-0·002	-0·003	-0·002	0·033	0·030	0·033
**diarrhea(SE)**	(0·002)	(0·002)	(0·002)	(0·001)	(0·001)	(0·001)	(0·005)	(0·007)	(0·005)
**p value**	0·079	0·137	0·067	0·044	0·027	0·044	<0·001	<0·001	<0·001
**No care sought for cough**	0·001	0·006	0·001	-0·001	0·001	-0·001	0·026	0·018	0·026
**(SE)**	(0·002)	(0·004)	(0·002)	(0·001)	(0·002)	(0·001)	(0·006)	(0·009)	(0·006)
**p value**	0·731	0·108	0·785	0·387	0·569	0·368	<0·001	0·042	<0·001
**High indoor pollution**	-0·002	-0·003	-0·002	-0·001	-0·001	-0·001	-0·005	0·005	-0·004
**(SE)**	(0·001)	(0·002)	(0·001)	(0·001)	(0·001)	(0·001)	(0·004)	(0·005)	(0·004)
**p value**	0·107	0·067	0·074	0·236	0·164	0·180	0·200	0·318	0·234
**No access safe drinking**	-0·001	-0·003	-0·001	0·001	0·000	0·001	0·021	0·021	0·022
**water (SE)**	(0·001)	(0·002)	(0·001)	(0·001)	(0·001)	(0·001)	(0·003)	(0·005)	(0·003)
**p value**	0·317	0·084	0·257	0·041	0·657	0·067	<0·001	<0·001	<0·001
**No access improved**	-0·001	-0·002	-0·001	-0·001	-0·002	-0·001	0·022	0·025	0·022
**sanitation (SE)**	(0·001)	(0·001)	(0·001)	(0·001)	(0·001)	(0·001)	(0·003)	(0·004)	(0·003)
**p value**	0·310	0·266	0·304	0·346	0·055	0·260	<0·001	<0·001	<0·001
**Inadequate antenatal**	-0·000	0·001	-0·001	0·000	0·000	0·000	0·023	0·009	0·023
**care (SE)**	(0·001)	(0·002)	(0·001)	(0·001)	(0·001)	(0·001)	(0·004)	(0·005)	(0·004)
**p value**	0·891	0·517	0·571	0·763	0·799	0·947	<0·001	0·090	<0·001
**No skilled birth attendant**	0·000	0·000	0·000	-0·001	-0·000	-0·001	0·034	0·022	0·033
**(SE)**	(0·002)	(0·002)	(0·002)	(0·001)	(0·001)	(0·001)	(0·004)	(0·006)	(0·004)
**p value**	0·880	0·832	0·770	0·081	0·724	0·129	<0·001	<0·001	<0·001
**Unsatisfied family**	-0·002	-0·002	-0·002	-0·002	-0·002	-0·002	0·006	0·008	0·007
**planning(SE)**	(0·001)	(0·002)	(0·001)	(0·001)	(0·001)	(0·001)	(0·003)	(0·004)	(0·003)
**p value**	0·037	0·143	0·105	0·004	0·002	0·010	0·025	0·055	0·021
**Maternal child marriage**	-0·003	-0·004	-0·003	-0·000	-0·000	-0·000	0·008	0·005	0·007
**(SE)**	(0·002)	(0·002)	(0·002)	(0·001)	(0·001)	(0·001)	(0·004)	(0·006)	(0·004)
**p value**	0·029	0·058	0·036	0·994	0·891	0·973	0·035	0·459	0·065
**No maternal education**	-0·002	-0·003	-0·002	-0·000	-0·001	-0·000	0·021	0·025	0·022
**(SE)**	(0·001)	(0·002)	(0·001)	(0·001)	(0·001)	(0·001)	(0·004)	(0·005)	(0·004)
**p value**	0·240	0·111	0·264	0·573	0·520	0·530	<0·001	<0·001	<0·001
**Low maternal height**	-0·004	-0·002	-0·004	-0·002	-0·001	-0·002	0·011	0·008	0·010
**(SE)**	(0·002)	(0·002)	(0·002)	(0·001)	(0·001)	(0·001)	(0·005)	(0·006)	(0·005)
**p value**	0·063	0·279	0·063	0·074	0·176	0·077	0·030	0·192	0·044
**Low maternal BMI**							0·010	0·011	0·011
**(SE)**							(0·004)	(0·005)	(0·004)
**p value**							0·005	0·030	0·005
**High maternal BMI**	0·014	0·012	0·014	0·005	0·004	0·005			
**(SE)**	(0·001)	(0·002)	(0·001)	(0·001)	(0·001)	(0·001)			
**p value**	<0·001	<0·001	<0·001	<0·001	<0·001	<0·001			
**Number of observations**	135,669	135,669	134,207	134,079	134,079	133,555	98,842	98,842	94,962

Part 2

BMI=body mass index (calculated as weight in kilograms divided by height in meters squared). ORT=oral rehydration therapy. All regressions are ordinary least squares. All specifications are ordinary least squares and include country-fixed effects as well as household and child/mother control variables. SEs are clustered at the PSU level.

© 2023 Büttner N et al. *JAMA Network Open*.

eTable 6. Association Between Economic Growth and Determinants of Malnutrition—Sanity Tests

	Delayed breastfeeding	No vitamin A suppl.	No iodized salt use	Incomplete vaccination	Recent infectious disease	No ORT given with diarrhea	No care sought for cough	High indoor pollution
**Unweighted**								
Log of per-head GDP	0·0025	-0·0015	-0·0328	0·0004	0·0019	-0·0004	0·0008	-0·0048
(SE)	(0·0003)	(0·0003)	(0·0016)	(0·0002)	(0·0002)	(0·0001)	(0·0001)	(0·0002)
p value	<0·001	<0·001	<0·001	0·062	<0·001	0·004	<0·001	<0·001
Number of observations	910,642	834,572	419,397	1,051,314	1,067,017	1,041,452	1,019,887	977,267
**Weighted**								
Log of per-head GDP	0·0032	-0·0015	-0·0550	-0·0005	0·0015	-0·0006	-0·0003	-0·0036
(SE)	(0·0003)	(0·0004)	(0·0014)	(0·0002)	(0·0003)	(0·0002)	(0·0001)	(0·0004)
p value	<0·001	<0·001	<0·001	0·021	<0·001	<0·001	0·061	<0·001
Number of observations	910,642	834,572	419,397	1,051,314	1,067,017	1,041,452	1,019,887	977,267
**Trimmed**								
Log of per-head GDP	0·0025	-0·0023	-0·0305	0·0001	0·0012	-0·0001	0·0008	-0·0051
(SE)	(0·0003)	(0·0004)	(0·0017)	(0·0002)	(0·0002)	(0·0001)	(0·0001)	(0·0002)
p value	<0·001	<0·001	<0·001	0·508	<0·001	0·368	<0·001	<0·001
Number of observations	883,445	824,240	414,444	1,016,255	1,048,261	1,005,715	876,281	940,003
**Instrumental Variable**								
Log of per-head GDP	0·0130	-0·0514	0·0138	-0·0130	0·0210	0·0038	0·0021	0·0085
(SE)	(0·0056)	(0·0037)	(0·0194)	(0·0054)	(0·0033)	(0·0018)	(0·0017)	(0·0063)
p value	0·019	<0·001	0·478	0·016	<0·001	0·038	0·207	0·177
Number of observations	910,642	833,883	419,397	1,050,158	1,065,511	1,040,450	1,019,229	972,927

Part 1

ORT=oral rehydration therapy. Data for per-head gross domestic product (GDP) were merged with Demographic and Health Survey data by survey year. All regressions are ordinary least squares, and the instrumental variable regressions are two-stage least squares. All specifications include country and survey-year fixed effects as well as household control variables. SEs are clustered at the PSU level. Coefficients for the log of per-head GDP represent a 5% increase in per-head GDP. In the instrumental variable regressions, we used the variable share of gross capital formation at present purchasing power parity (investment share of GDP) from the Penn World Tables 10.0, with a 5-year lag as an instrument for the log of the per-head GDP.

© 2023 Büttner N et al. *JAMA Network Open*.

	No access safe drinking water	No access improved sanitation	Unsafe stool disposal	Inade-quate antenatal care	No skilled birth attendant	Unsatis-fied family planning	Maternal child marriage	No maternal educ.	Low maternal height	Low maternal BMI
**Unweighted**										
Log of per-head GDP	0·0018	-0·0021	-0·0096	-0·0027	-0·0004	0·0016	-0·0003	-0·0001	-0·0008	-0·0009
(SE)	(0·0003)	(0·0003)	(0·0005)	(0·0002)	(0·0002)	(0·0003)	(0·0002)	(0·0002)	(0·0001)	(0·0002)
p value	<0·001	<0·001	<0·001	<0·001	0·082	<0·001	0·149	0·522	<0·001	<0·001
Number of observations	1,082,733	1,094,647	753,504	970,018	1,077,132	637,120	1,035,846	1,138,568	902,576	826,307
**Weighted**										
Log of per-head GDP	-0·0027	-0·0027	-0·0101	-0·0006	0·0006	0·0007	0·0009	-0·0000	-0·0008	-0·0009
(SE)	(0·0005)	(0·0004)	(0·0007)	(0·0003)	(0·0003)	(0·0004)	(0·0003)	(0·0003)	(0·0001)	(0·0002)
p value	<0·001	<0·001	<0·001	0·048	0·090	0·084	0·003	0·972	<0·001	<0·001
Number of observations	1,082,733	1,094,647	753,504	970,018	1,077,132	637,120	1,035,846	1,138,568	902,576	826,307
**Trimmed**										
Log of per-head GDP	0·0014	-0·0018	-0·0076	-0·0027	-0·0005	0·0017	-0·0003	0·0003	-0·0006	-0·0003
(SE)	(0·0003)	(0·0003)	(0·0005)	(0·0002)	(0·0003)	(0·0003)	(0·0002)	(0·0002)	(0·0001)	(0·0002)
p value	<0·001	<0·001	<0·001	<0·001	0·039	<0·001	0·200	0·170	<0·001	0·070
Number of observations	1,052,950	1,045,435	733,816	940,669	1,047,046	543,821	1,005,954	1,107,621	877,445	753,075
**Instrumental Variable**										
Log of per-head GDP	-0·0748	-0·0513	-0·0375	-0·0036	0·0981	0·4495	0·0115	0·0166	0·0010	0·0010
(SE)	(0·0099)	(0·0052)	(0·0033)	(0·0028)	(0·0091)	(0·3455)	(0·0039)	(0·0039)	(0·0011)	(0·0017)
p value	<0·001	<0·001	<0·001	0·204	<0·001	0·193	0·003	<0·001	0·373	0·551
Number of observations	1,077,165	1,089,079	753,504	964,891	1,075,934	637,120	1,034,645	1,136,720	902,576	826,307

Part 2

BMI=body mass index (calculated as weight in kilograms divided by height in meters squared). Data for the per-head gross domestic product (GDP) were merged with Demographic and Health Survey data by survey year. All regressions are ordinary least squares, and the instrumental variable regressions are two-stage least squares. All specifications include country and survey-year fixed effects as well as household control variables. SEs are clustered at the PSU level. Coefficients for the log of per-head GDP represent a 5% increase in per-head GDP. In the instrumental variable regressions, we used the variable share of gross capital formation at present purchasing power parity (investment share of GDP) from the Penn World Tables 10.0, with a 5-year lag as an instrument for the log of the per-head GDP.

© 2023 Büttner N et al. *JAMA Network Open*.

eTable 7. Association Between Economic Growth and Malnutrition—Shorrocks Decomposition

Contribution of explanatory variables to pseudo R-squared (%)	Stunting	Wasting	Under-weight	Over-weight	Obesity	Dietary Diversity Failure
Log of per-head GDP	5.28	0.65	2.21	1.28	0.94	6.75
Control variables	60.09	7.63	41.71	49.02	47.27	58.41
Year fixed Effects	6.11	24.30	11.66	13.67	17.09	7.93
Country fixed effects	28.53	67.43	44.42	36.03	34.71	26.91
Pseudo R-squared	0.08	0.08	0.12	0.08	0.08	0.16

Each column refers to one regression decomposition. Data for the per-head gross domestic product (GDP) were merged with Demographic and Health Survey data by survey year. All specifications include country and survey-year fixed effects as well as household and child/mother control variables.

eTable 8. Association Between Determinants and Indicators of Malnutrition—Shorrocks Decomposition

Contribution of explanatory variables to pseudo R-squared (%)	Stunting	Wasting	Under-weight	Over-weight	Obesity	Dietary Diversity Failure
Contributing factors	38.26	18.62	31.46	18.12	19.54	24.69
Control variables	29.86	5.24	23.53	37.94	32.22	39.40
Country fixed effects	31.88	76.14	45.01	43.94	48.24	35.92
Pseudo R-squared	0.08	0.10	0.14	0.09	0.09	0.17

Each column refers to one regression decomposition. All specifications include country-fixed effects as well as household and child/mother control variables.

© 2023 Büttner N et al. *JAMA Network Open*.

eTable 9. Association Between Economic Growth and Determinants of Malnutrition—Shorrocks Decomposition

Contribution of explanatory variables to pseudo R-squared (%)	Delayed breastfeeding	No vitamin A suppl.	No iodized salt use	Incomplete vaccination	Recent infectious disease	No ORT given with diarrhea	No care sought for cough	High indoor pollution
Log of per-head GDP	2.76	5.38	11.16	0.87	9.26	4.43	5.66	19.11
Control variables	1.90	7.85	6.72	16.10	1.35	9.19	5.83	38.14
Year fixed Effects	13.02	25.14	22.10	12.67	27.41	26.59	35.45	6.06
Country fixed effects	82.32	61.63	60.02	70.36	61.97	59.79	53.06	36.69
Pseudo R-squared	0.05	0.06	0.24	0.05	0.06	0.05	0.08	0.64

Part 1

Each column refers to one regression decomposition. Data for the per-head gross domestic product (GDP) were merged with Demographic and Health Survey data by survey year. All specifications include country and survey-year fixed effects as well as household control variables.

Contribution of explanatory variables to pseudo R-squared (%)	No access safe drinking water	No access improved sanitation	Unsafe stool disposal	Inade-quate antenatal care	No skilled birth attendant	Unsatis-fied family planning	Maternal child marriage	No maternal educ.	Low maternal height	Low maternal BMI
Log of per-head GDP	6.13	4.35	3.63	7.62	9.07	12.13	8.04	6.41	5.61	1.08
Control variables	35.85	52.99	29.61	35.55	35.67	12.20	34.01	35.56	13.25	15.22
Year fixed Effects	14.96	11.17	15.67	8.79	16.09	37.61	12.22	6.67	16.71	18.87
Country fixed effects	43.07	31.48	51.09	48.05	39.17	38.06	45.73	51.37	64.43	64.82
Pseudo R-squared	0.18	0.36	0.15	0.14	0.27	0.18	0.10	0.28	0.14	0.12

Part 2

Each column refers to one regression decomposition. Data for the per-head gross domestic product (GDP) were merged with Demographic and Health Survey data by survey year. All specifications include country and survey-year fixed effects as well as household control variables.

© 2023 Büttner N et al. *JAMA Network Open*.

eFigure 1. Association Between Determinants and Indicators of Malnutrition—Standard Model (15 Determinants)

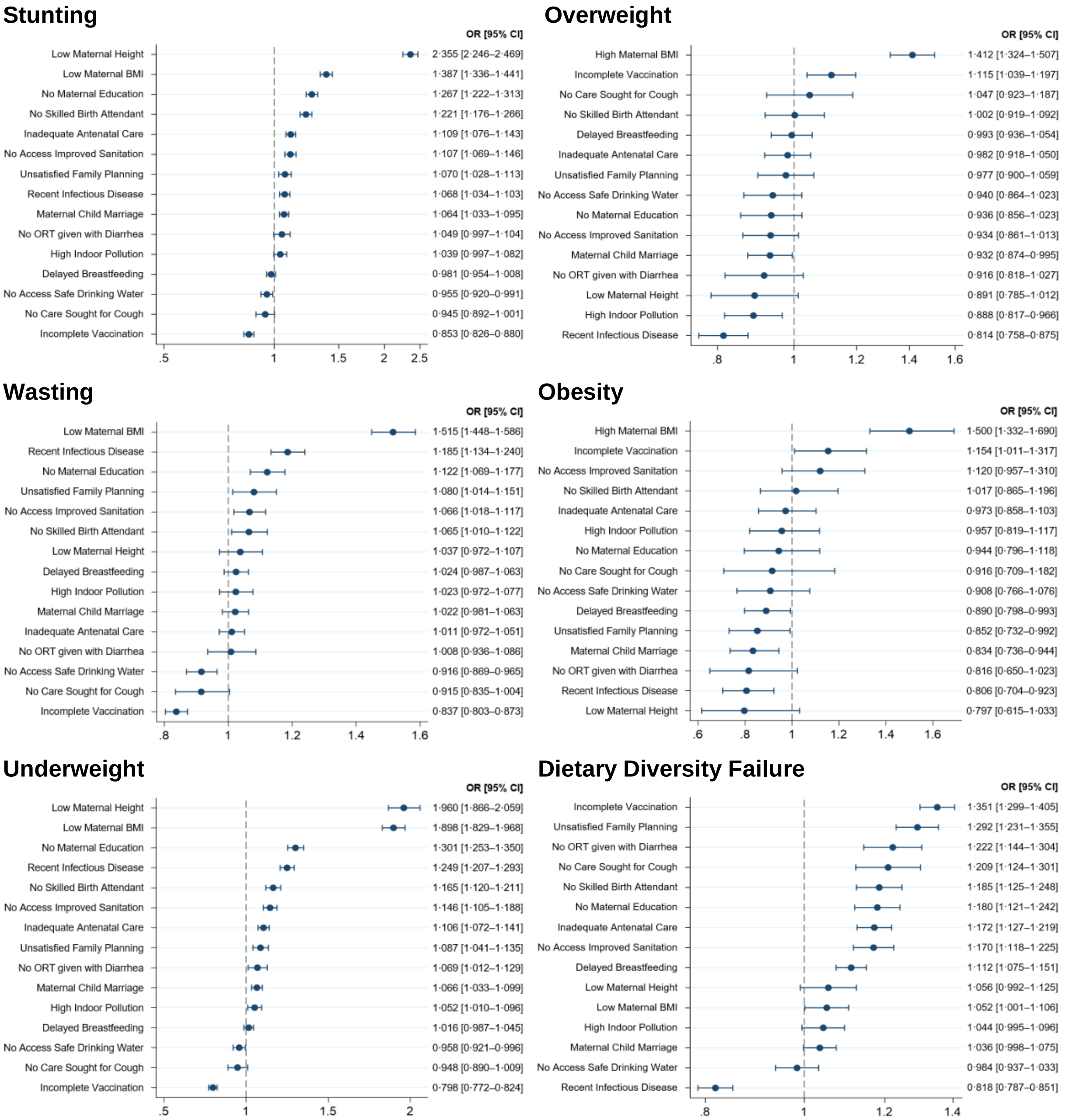

Circles represent point estimates of odds ratios associated with the various measures of malnutrition. Horizontal bars indicate 95% confidence intervals. BMI=body mass index (calculated as weight in kilograms divided by height in meters squared). ORT=oral rehydration therapy. N=135,310; Countries=56 (stunting). N=135,669; Countries=56 (wasting). N=135,567; Countries=56 (underweight). N=135,669; Countries=56 (overweight). N=134,079; Countries=55 (obesity). N=98,842; Countries=51 (dietary diversity failure).

© 2023 Büttner N et al. *JAMA Network Open*.

Figure 2. Association Between Determinants and Indicators of Malnutrition—Extended Model (18 Determinants)

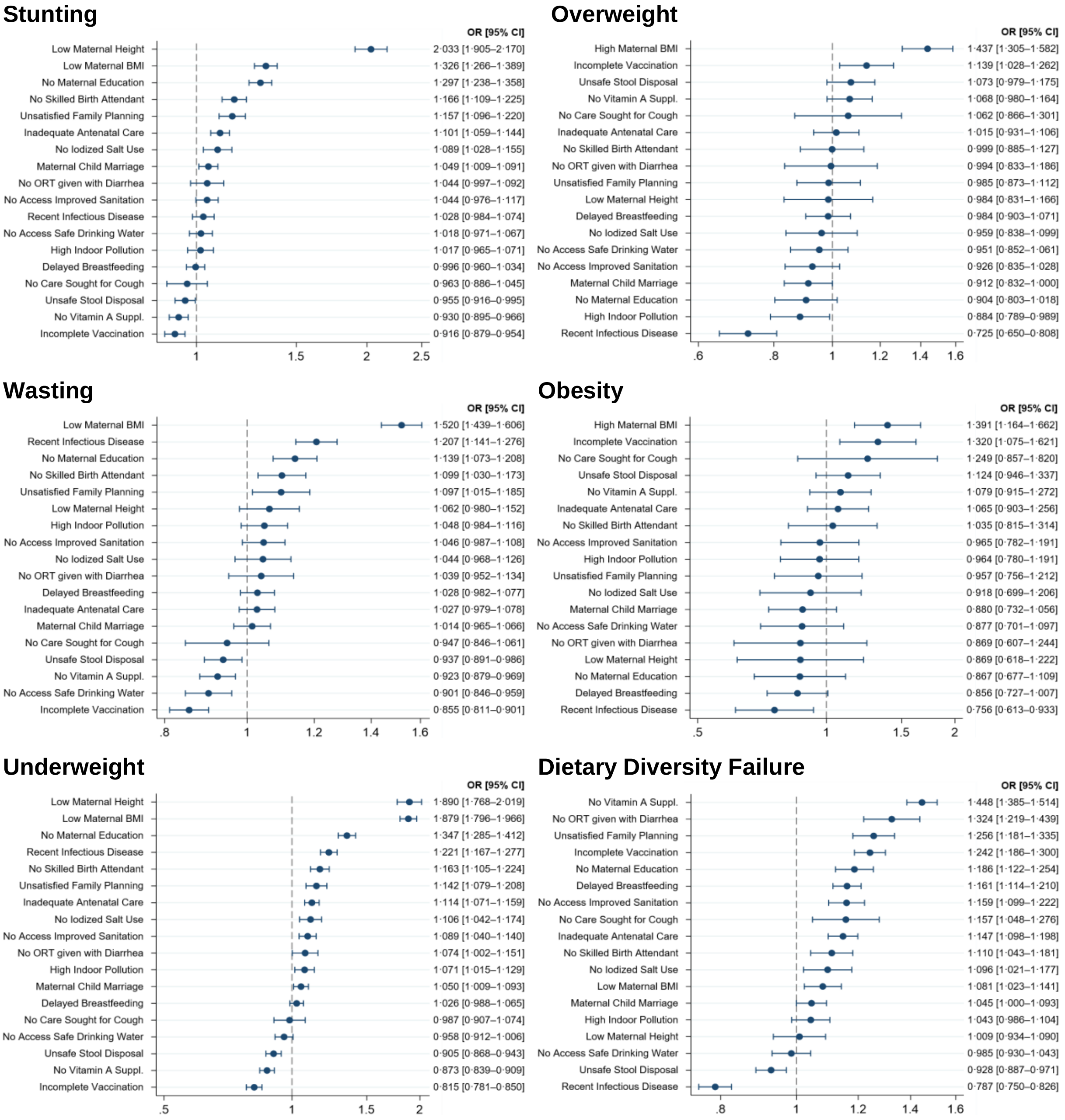

Circles represent point estimates of odds ratios associated with the various measures of malnutrition. Horizontal bars indicate 95% confidence intervals. BMI=body mass index (calculated as weight in kilograms divided by height in meters squared). ORT=oral rehydration therapy. N=72,411; Countries=36 (stunting). N=72,467; Countries=36 (wasting). N=72,411; Countries=36 (underweight). N=72,467; Countries=36 (overweight). N=72,467; Countries=36 (obesity). N=73,262; Countries=36 (dietary diversity failure).

© 2023 Büttner N et al. *JAMA Network Open*.

eFigure 3. Associations Between Per-Head GDP and Contributing Factors of Malnutrition

Immediate determinants of malnutrition

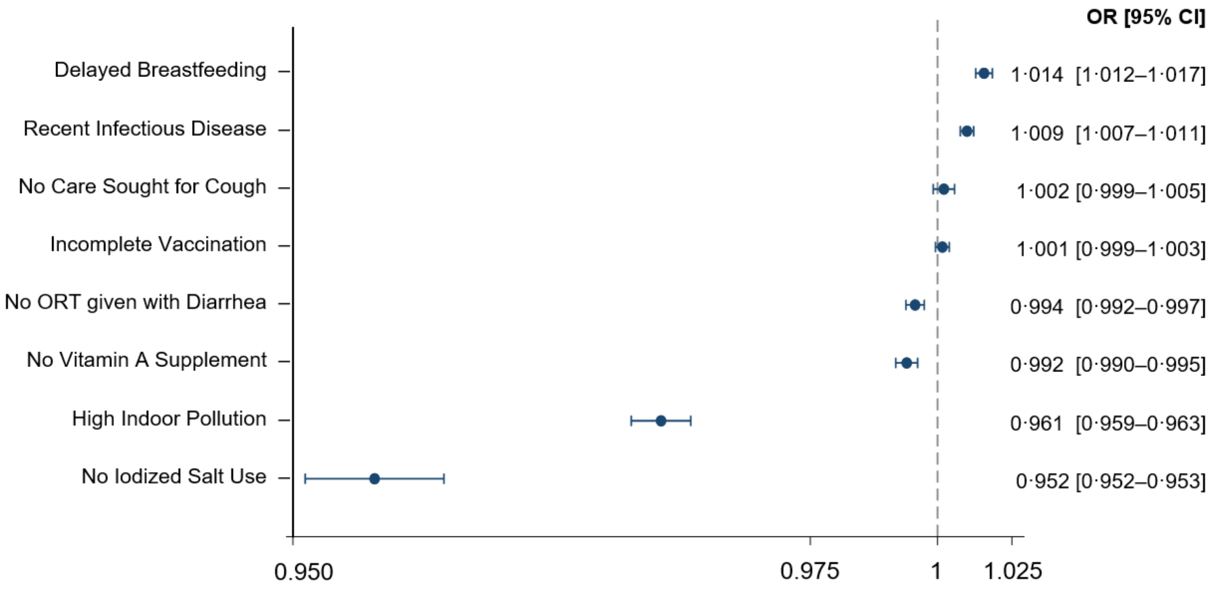

Underlying determinants of malnutrition

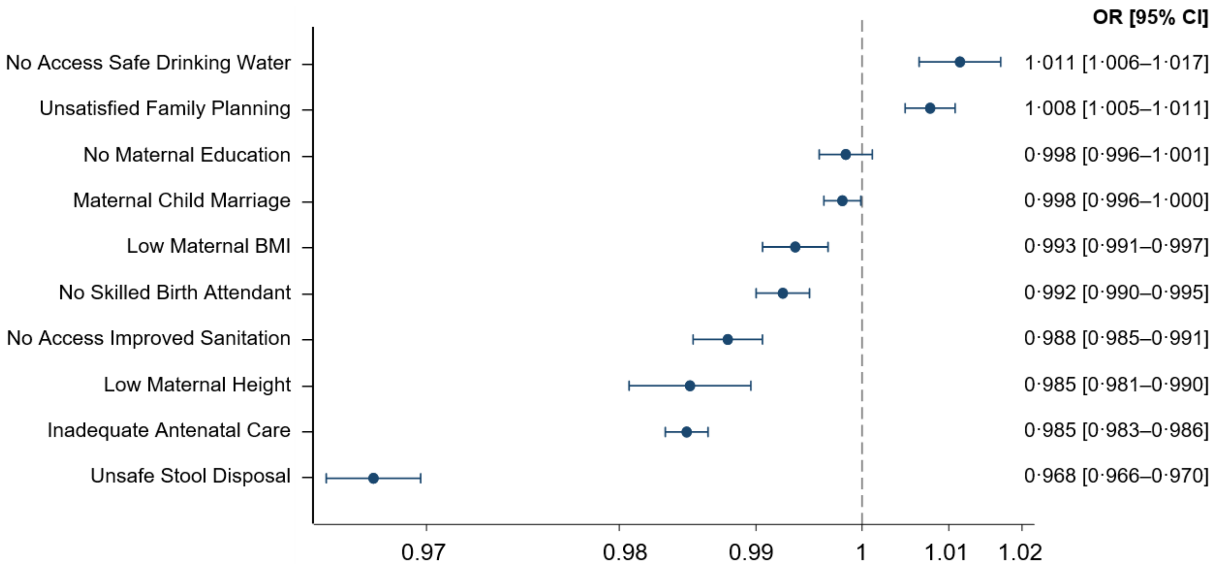

Circles represent point estimates of odds ratios associated with immediate determinants of malnutrition (a) and underlying determinants of malnutrition (b). Horizontal bars indicate 95% confidence intervals. GDP=gross domestic product. ORT=oral rehydration therapy. N=834,572 (no Vitamin A supplementation), N= 910,642 (delayed breastfeeding initiation), N= 1,067,017 (incidence of infectious diseases), N= 1,051,314 (incomplete course of vaccination), N= 1,019,887 (no care seeking for suspected pneumonia despite cough), N= 1,041,452 (no consumption of oral rehydration solution despite diarrhea), N= 977,267 (high indoor pollution through solid cooking fuels), N= 419,397 (no iodized salt use). N= 637,120 (unsatisfied family planning needs), N= 1,035,846 (maternal child marriage), N= 1,138,568 (no maternal education), N= 1,077,132 (no skilled birth attendant), N= 1,082,733 (no access to safe drinking water), N= 826,307 (low maternal BMI), N= 902,576 (low maternal height), N= 1,094,647 (no access to improved sanitation facilities), N= 970,018 (inadequate antenatal care), N= 753,504 (unsafe practices for child’s stool disposal).

© 2023 Büttner N et al. *JAMA Network Open*.
