# Supplementary material for: Economic Growth and Childhood Malnutrition in Low- and Middle-Income Countries
Source: JAMA Netw Open. 2023 Nov 9;6(11):e2342654. doi: 10.1001/jamanetworkopen.2023.42654 (PMC10636637; doi:10.1001/jamanetworkopen.2023.42654)
Supplement: Supplement 2. — Data Sharing Statement [file jamanetwopen-e2342654-s002.pdf]

Data Sharing Statement

Büttner. Economic Growth and Childhood Malnutrition in Low- and Middle-Income Countries. *JAMA Netw Open*. Published November 09, 2023. doi:10.1001/jamanetworkopen.2023.42654

Data
Data available: No

Additional Information
Explanation for why data not available: The data used in this study is publicly available.
